# The search for molecular mimicry in proteins carried by extracellular vesicles secreted by cells infected with *Plasmodium falciparum*

**DOI:** 10.1080/19420889.2021.1972523

**Published:** 2021-09-08

**Authors:** Vinicio Armijos-Jaramillo, Andrea Mosquera, Brian Rojas, Eduardo Tejera

**Affiliations:** a Carrera de Ingeniería en Biotecnología, Facultad de Ingeniería y Ciencias Aplicadas, Universidad de Las Américas, Quito, Ecuador; b Grupo de Bio-Quimioinformática, Universidad de Las Américas, Quito, Ecuador

**Keywords:** *Plasmodium falciparum*, molecular mimicry, extracellular vesicles, *in-silico* analysis, structural resemblance, moonlighting protein

## Abstract

Red blood cells infected with *Plasmodium falciparum* secrete extracellular vesicles in order to facilitate the survival and infection of human cells. Various researchers have studied the composition of these extracellular vesicles and identified the proteins contained inside. In this work, we used that information to detect potential *P. falciparum* molecules that could be imitating host proteins. We carried out several searches to detect sequences and structural similarities between the parasite and host. Additionally, the possibility of functional mimicry was explored in line with the potential role that each candidate can perform for the parasite inside the host. Lastly, we determined a set of eight sequences (mainly moonlighting proteins) with a remarkable resemblance to human proteins. Due to the resemblance observed, this study proposes the possibility that certain *P. falciparum* molecules carried by extracellular vesicles could be imitating human proteins to manipulate the host cell’s physiology.

## Introduction

Malaria is a tropical disease that has a huge impact on the world, with a point estimation of 409,000 deaths worldwide in 2019 [1]. The sickness is transmitted via an infectious bite from a female *Anopheles* mosquito. This insect carries the *Plasmodium* spp. and infects the host through a bite that releases the parasite into the bloodstream [2]. Six species have the ability to infect humans and produce malaria: *Plasmodium falciparum, Plasmodium vivax, Plasmodium ovale wallickeri, Plasmodium ovale curtisi, Plasmodium malariae*, and *Plasmodium knowlesi*. Of these, *P. falciparum* causes the more deadly forms of malaria [3].

The parasite-infected red blood cells (iRBC) secrete nanometric particles known as extracellular vesicles that facilitate their survival and at the same time allow cellular infection. This is mainly through the immunomodulation produced in the host and the communication facilitated between parasites [4]. Extracellular vesicles (EVs) can also be found in normal cellular processes, as they are fragments that detach almost spontaneously from the plasma membrane vials [5]. Pathogens manipulate the cells into releasing EVs in order to modulate the host’s immune system to promote survival. This release increases when cells are subjected to a variety of stress conditions [6]. EVs have the ability to transfer biological information between donor and recipient cells, either closely or remotely, and are therefore considered vehicles capable of protecting and delivering information through cells [7].

Studies conducted on EVs during *P. falciparum* infection show that they are capable of promoting different alterations in the host, such as neutrophil activation, secretion of anti-inflammatory and pro-inflammatory cytokines, induction of gametogenesis, and the incorporation of DNA plasmids. Studies also show that the protozoon uses EVs for parasite-to-parasite and host-to-parasite communication [8]. Based on these findings, the presence of EVs was suggested as a potential marker for the presence of *P. falciparum* inside the host. Additionally, a recent proteomic analysis identified several proteins that may be involved in red-blood-cell invasion by the molecular mimicking of host molecules, specifically the PEXEL peptide, which is exported by *Plasmodium*, and also the ring-exported protein 2 (Rex2). Both show molecular similarity to the RAC2 protein family of *Homo sapiens* [9].

The ability of a molecule to resemble another in structure or function is known as molecular mimicry [10]. In the case of species whose changes toward mimicry increase their fitness or the sequence conservation among distantly related species, the similarity between two molecules could be produced by adaptation (adaptive mimicry). Another case of similarity could be obtained through convergent evolution, a phenomenon called consequential mimicry [11]. A typical example of this kind of event occurs when molecules – usually proteins, carbohydrates or deoxyribonucleic acid – shared between the pathogen and the host cause a cross-reaction in the presence of an active immune response [12].

In this work, we explore the potential of *P. falciparum* proteins (carried by EVs during a malaria infection) to imitate human molecules. To the best of our knowledge, this is the first time that these kinds of proteins have been tested for mimetic ability. To achieve this goal, several steps of searches and filters were applied to find the most plausible candidates. A molecular mimicry scenario could unveil several aspects of the *Plasmodium* invasion, such as the immune evasion or intercellular communication performed by the parasites. Additionally, molecular mimicry scenarios could be responsible for producing some of the rare post-infection effects, like post-malaria neurological syndrome [13]. This was observed in a similar way in the Guillain-Barré syndrome produced following a *Campylobacter jejuni* infection [14].

## Methods

### Extracellular vesicle proteins: search and downloading

A literature search was carried out to determine reported proteins of *Plasmodium falciparum* in extracellular vesicles. The search criteria focused on bibliographic sources, where proteomics of extracellular vesicles from infected human blood cells had been performed. We retrieved all PubMed (https://pubmed.ncbi.nlm.nih.gov/) articles fulfilling the following search criteria: (plasmodium falciparum) AND (extracellular vesicles) AND (proteomic). All articles written up to January 2021 were included. With these criteria, only 7 manuscripts were found (Correa *et al* [9]., Jiang *et al* [15]., Tiberti *et al* [16]., Antwi-Baffour *et al* [17]., Sampaio *et al* [18]., Abdi *et al* [19]., and Mantel et al [4].). Each article discovered was manually analyzed and the following was observed. 1) In Correa et al [9]., the proteomic comparison was made between EVs with low and high parasitemia; no raw data of proteomic analysis was published. 2) In Jiang et al [15]., no proteomic analysis was carried out. 3) In Tiberti et al [16]., the analysis was not performed with *Plasmodium falciparum* but *Plasmodium Berghei*. 4) In Antwi-Baffour et al [17]., a proteomic analysis was carried out and a list of proteins was provided but no raw data was presented, nor was there any information regarding the confidence of the annotated proteins. Considering these aspects only three manuscripts were further considered in the analysis: Sampaio et al [18]., Abdi et al [19]., and Mantel et al [4].

**Figure 1.**
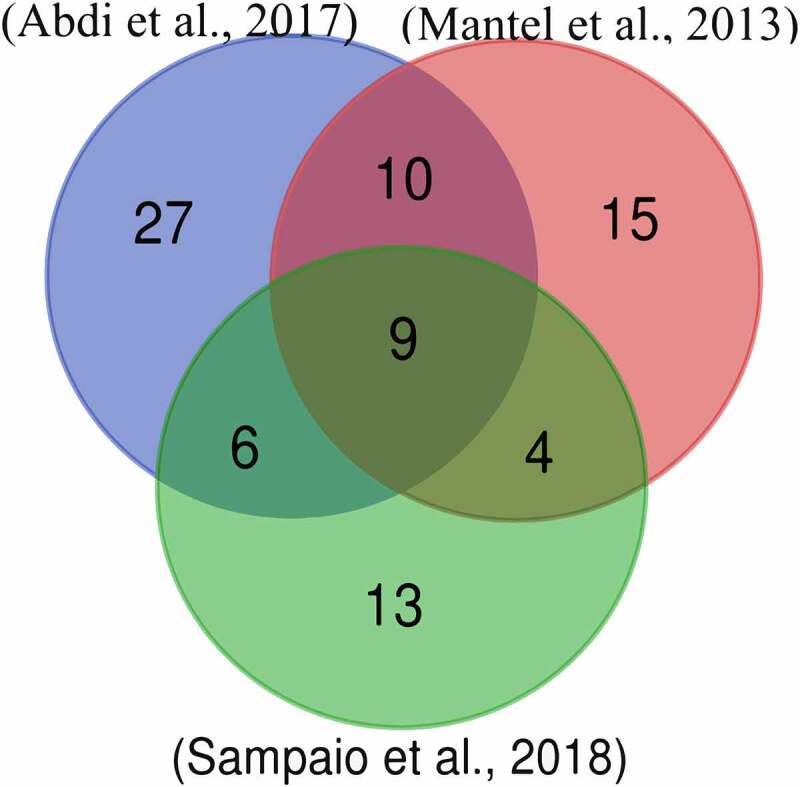
Venn diagram of *Plasmodium falciparum* 3D7 proteins reported in extracellular vesicles during parasite infection according to several authors

In order to homogenize information and confidence from the raw data of selected studies, from the already significant proteins identified we only included those with a minimum value of unique peptide count higher than 3. Then, we identified the sequences shared by two or more publications in order to determine *P. falciparum* proteins observed in human vesicles by independent authors (Figure 1). All these sequences were downloaded from the UniProt (https://www.uniprot.org/) database. In addition, the human proteome (GRCh38 version) was downloaded from NCBI to perform BLASTp searches, as described in the section below.

### BLAST searches and tertiary structure recovery

To detect similarities between human proteins and the *P. falciparum* sequences observed in vesicles, we performed a BLASTp search [20] using the human proteome (GRCh38) as a database and sequences from Supplementary Table 2 as a query. The results were filtered by selecting query proteins with a percentage of identity greater than at least 40% and a coverage of 70%. The sequences that passed this filter were considered for the following steps. Moreover, pairs of sequences (putative imitator and imitated) were aligned using MAFFT [21]. The domains and motif prediction for the candidates’ sequences was performed with InterProScan [22].

The tertiary structure was downloaded from the PDB (Protein Data Bank) [23] database (https://www.rcsb.org/). For the sequences without crystalized structures in PDB, the tool Phyre2 [24] (http://www.sbg.bio.ic.ac.uk/phyre2/) was used to predict the tertiary structures.

### Structural comparisons

The pairs of 3D structures (*P. falciparum –* human proteins) were superimposed using Pymol 2.4 [25]. The same program was used to calculate the root-mean-square deviation (RMSD) between structures and quantify the structural similarities of each protein pair. In order to detect structural similarities between candidates and proteins deposited in the PDB, we used the DALI server [26] (http://ekhidna2.biocenter.helsinki.fi/dali/).

### Phylogenetic tree reconstruction

In order to determine whether the candidate proteins of *P. falciparum* came from horizontal transfer events, phylogenetic trees were reconstructed to clarify their evolutionary origin. BLAST searches were performed to detect putative homologue sequences. For that purpose, we used the human and *P. falciparum* candidate proteins as a query to perform a BLASTp search vs the NCBI’ NR database. Then, we collected the 20 best results and deleted duplicate sequences (pairs of sequences with 100% of identity and coverage). With these sequences, multiple sequence alignments were formed with MAFFT (default options) and then the trees were reconstructed using MEGA X [27]. For the three reconstructions, we used the tool included in MEGA to find the best protein model for each alignment and then employed this information to reconstruct the trees through the maximum likelihood algorithm.

## Results

We searched for studies that identified *Plasmodium* proteins inside human vesicles during the parasite infection. Three articles were selected according to the aforementioned criteria [4,18,19]. Supplementary Table 1 shows the gene names that correspond to the sequences recovered from each study. This information was used to make a Venn diagram (Figure 1) to determine which proteins coincided among the studies. The study of Adbi et al [19]. reported the largest number of proteins (52), followed by Mantel et al [4]. (38), and Sampaio et al [18]. (32). In all three experiments, similar procedures were utilized for vesicle isolation and all of them used a nano-LC as a chromatographic method for peptide separations after trypsin digestion. In Mantel et al [4]., the mass spectrometer was a Q-Exactive compared to Orbitrap in the other two studies. Two important differences between the studies are as follows. 1) In Abdi et al [19]., the plasmodium was extracted from a patient with cerebral malaria. Once isolated, it was adapted to *in vitro* culture and grown over a short period of time (70 cycles). This is a different approach to other studies where the 3D7 isolate was used. 2) In Sampaio et al. [18], only the topmost intense ion was further fragmented in contrast to the top 12 or 15 ions in the other two studies. These differences could possibly be related to the greater similarity between Abdi et al. [19] and Mantel et al [4]., as opposed to Sampaio et al [18].

We observed nine proteins identified by three independent research teams, so these were considered the most likely molecules to travel in vesicles during malaria development. Twenty further sequences were detected in two of the three studies (Supplementary Table 2). The 29 proteins found at the intersections of the Venn diagram were chosen as candidates for this study. The proteins found in at least two of three studies were further considered for analysis.

**Figure 2.**
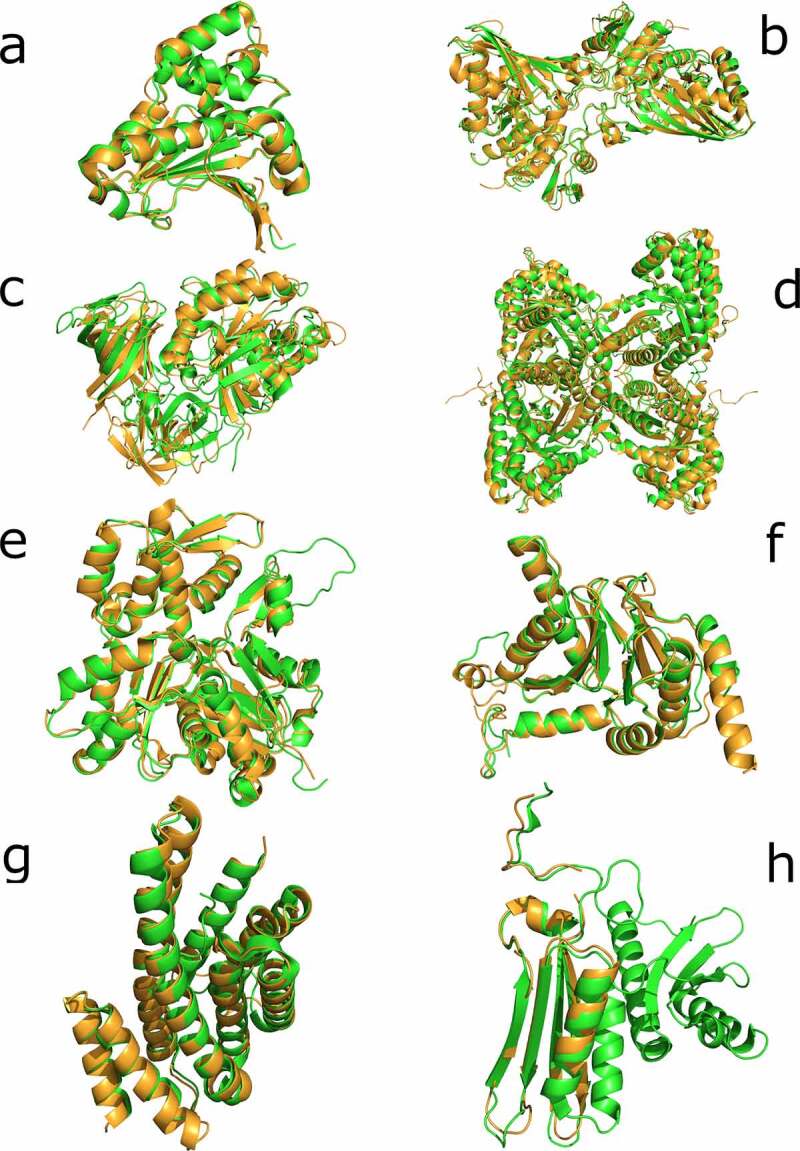
Superimposition of candidate proteins. (a) Heat shock protein 90, 6CEO (*Homo sapiens*) with 3K60 (*P. falciparum*). (b) Glyceraldehyde-3-phosphate dehydrogenase, 3GPD (*Homo sapiens*) with 1YWG (*P. falciparum*). (c) Elongation factor 1-alpha, 3C5J (*Homo sapiens*) with the homology predicted structure for (*P. falciparum*). (d) Fructose-aldose bisphosphate, 5LN3 (*Homo sapiens*) with 6MUX (*P. falciparum*). (e) Actin-1, 5 JLH (*Homo sapiens*) with 6I4K (*P. falciparum*). (f) Proteasome subunit alpha, 6REY (*Homo sapiens*) with 6MUW (*P. falciparum*). (g) 14-3-3 proteins, 3UVW (*Homo sapiens*) with the homology predicted structure for *P. falciparum*. (h) 40S ribosomal protein S3, 6YBS chain K (*Homo sapiens*) with 6OKK chain D (*P. falciparum*). The human proteins are represented in green and the *P. falciparum* proteins in light brown

From these 29 sequences, nine showed similarities with human proteins (more than 40% of similarity and 70% of query coverage – Supplementary Table 3). Additionally, we decide to analyze two sequences (PF3D7_0818900 and PF3D7_0708400) with a low coverage but high similarity as potential candidates of partial mimicry. Given this similarity, we considered all these sequences to be the most plausible candidates for performing molecular mimicry in humans. Then, we obtained the structure of these 11 sequences and their most similar pairs in humans (see Methods). We performed a superimposition between each pair of structures and then calculated the RMSD, the data for which is displayed in Supplementary Table 4. We noted that three of the five proteins with predicted structures (PF3D7_0818900, PF3D7_1117700, and PF3D7_0917900) were calculated with their human counterpart protein structure as a template for the tertiary reconstruction. For that reason, a direct comparison through RMSD is unreliable. We focused on the candidates with crystallized structures and on PF3D7_1357100 and PF3D7_0818200, the latter of which does not use human proteins as templates for the tertiary reconstruction (Figure 2). With the remaining sequences of *P. falciparum* (PF3D7_1462800, PF3D7_1357100, PF3D7_0818200, PF3D7_1246200, PF3D7_0727400, PF3D7_0708400, PF3D7_1444800, and PF3D7_1465900), we used the DALI web server to perform a search in PDB and recover the most similar structural results available for humans in the database. We expected to find the same proteins observed in the BLAST searches, but only in PF3D7_1444800 and PF3D7_0818200 did the best result in PDB coincide with the best BLAST result (Table 1).

**Table 1.** Dali best results for *P. falciparum* molecular mimicry candidates vs human proteins

Predicted function	UniProt gene name *P. falciparum*	PDB ID *P. falciparum*	Best DALI result in humans	% of identity	RMSD
Glyceraldehyde-3-phosphate dehydrogenase	PF3D7_1462800	1YWG	6YND	64	0.8
Elongation factor 1-alpha	PF3D7_1357100	Predicted structure	3J5Y	37	5.5
Actin-1	PF3D7_1246200	6I4K	4FO0	22	2.0
Proteasome subunit alpha type	PF3D7_0727400	6MUW	6RGQ	46	1.5
Heat shock protein 90	PF3D7_0708400	3K60	6CEO	72	1.7
14-3-3 protein I	PF3D7_0818200	Predicted structure	3UBW	60	1.2
Fructose-bisphosphate aldolase	PF3D7_1444800	2PC4	1XFB	57	1.1
40S ribosomal protein S3	PF3D7_1465900	6OKK	5UBA	13	3.6

In the phylogenetic analysis, none of the reconstructed trees showed evidence of horizontal gene transfer for the candidates (Supplementary Figures 1–8). These analyses also helped to determine the conservation level of the protein families of each candidate. In the six phylogenies reconstructed, a species tree-like topology was observed, suggesting a high level of conservation for each family.

## Discussion

In this work, we explored the hypothesis that *P. falciparum* proteins carried through extracellular vesicles (EVs) could have a mimetic role that is potentially relevant for the development of malaria infection. In order to find the most plausible candidates to achieve this role, we explored and applied several steps of search and comparison in proteins found in EVs during *P. falciparum* infection in human blood cells. As a result, we propose eight proteins of *P. falciparum* with molecular mimicry potential.

We found three studies that identified *P. falciparum* proteins inside EVs by experimental procedures [4,18,19]. From these, we selected only sequences identified by at least two independent experiments. With this criterion, we guaranteed molecules transported by EVs during the parasite infection. The Venn diagram indicates that from 84 proteins, 29 were found in at least two studies.

With the chosen proteins, we decided to test the possibility of adaptive mimicry through sequence similarity (BLAST searches) and phylogenetic reconstructions. BLAST searches against human proteome exposed several proteins with high similarities to their human pairs. However, the phylogenetic analysis did not reveal any sign of horizontal transference origin; instead, species tree-like topologies were observed. Adaptive molecular mimicry can be achieved among distantly related species by sequence conservation or by horizontal gene transfer [11]. Moreover, sequence conservation could also imply the functional constraint of a sequence in both species. Reinforcing this possibility, we found shared motifs and domains by putative imitator and imitated candidates (Supplementary Table 5). Nevertheless, without functional experimental, it is hard to distinguish between the two alternatives for our candidates.

Several cases of molecular mimicry have been reported in the past without sequence similarities but with structural resemblances [28,29]. To test this possibility, we compared the 3D conformation of our candidates in order to determine the level of structural similarity between potential imitated and imitating molecules. The structural similarities can be observed in Figure 2 and for all the candidates, the superimpositions are remarkable. Said similarities alone do not imply functional molecular mimicry; other factors like subcellular space co-localization with the imitated molecule or the correct expression timing should be considered [30]. For the candidates, the opportunity to be carried by EVs makes them able to locate in several subcellular compartments at different times, allowing them to imitate several molecules. In addition to structural similarities and colocation, the function of the candidates should be analyzed to predict their potential role as mimetic molecules.

The candidate annotated as actin-1 (PF3D7_1246200) is part of a conserved group of proteins in Apicomplexa that has marked differences to mammals. This protein in *Plasmodium* spp. has been associated with the invasion of red blood cells, but also with endocytosis, secretion, and antigenic variation [31]. The role of actin in vesicular trafficking is widely known [32], but could this function explain the presence of *P. falciparum* actin inside human EVs? Does *P. falciparum* actin-1 help in EV trafficking during malaria infection? The structural resemblances with human actin are remarkable but they are not identical. However, PF3D7_1246200 is also similar to the actin-related protein Arp8 (PDB id. 4FO0), which is in agreement with the DALI search (Table 1). Arp8 proteins play a role in DNA repairing [33]. Our experiments have not elucidated the potential function and potentiality of a mimetic role, but rather have opened up an interesting field that could be investigated further with the intriguing presence of this molecule inside EVs.

Glyceraldehyde-3-phosphate dehydrogenase (GAPDH) has different functions in addition to its role in glycolysis. This molecule can take part in membrane transport and fusion, microtubule assembly, nuclear RNA export, translation control, and DNA replication and repair, among other tasks. These functions depend on its location and posttranslational modifications [34]. GAPDH is one of the molecules that has been denominated as moonlighting proteins because of its ability to perform more than one physiologically relevant biochemical or biophysical function [35]. The GAPDH found in our candidates (PF3D7_1462800) is remarkably similar to its human pair, and its location inside an EV gives it the opportunity to locate in several human subcellular spaces. Can the *P. falciparum* GAPDH interfere with the physiology of human cells? The plasticity of these kinds of molecules makes this hypothesis plausible.

Fructose-bisphosphate aldolase is a central enzyme of glycolysis, but it may perform other non-related functions, and in that sense, this enzyme can be considered a moonlighting protein. In *Plasmodium* spp., it can connect the actin filaments to thrombospondin-related anonymous protein (TRAP) in order to conduct the motor force through the *Plasmodium* surface [36]. Our study uncovered a fructose-bisphosphate aldolase (PF3D7_1444800) that was highly similar in structure to the human aldolase 1XFB. In humans, alongside its role in glycolysis, aldolase has been reported in the interaction between the sperm head and the zona pellucida [37]. Despite the structural similarities between aldolases in both species and the multifunctionality of the molecule, the role of PF3D7_1444800 in EVs is not evident and its ability to perform molecular mimicry remains uncertain.

A third moonlighting protein was identified in the list of mimetic candidates (PF3D7_0708400): heat-shock protein 90 (HSP90). These kinds of molecules have several functions, for example they facilitate protein maturation, keeping proteins in functional conformations, and check for misfolded proteins [38]. In *Plasmodium*, HSP90 has a role in transporting proteins from the parasite cytoplasm to erythrocyte [39]. Additionally, in *P. falciparum*, HSP90 is coupled with R2TP to drive several cellular processes, such as cell division [40]. However, the role of this molecule in EVs is unknown. In animals, HSP90 transported by EVs was detected in mice with alcoholic liver disease, mediating the activation of macrophages [41]. The structural similarities between 3K60 and 6CEO (*P. falciparum* and *H. sapiens* HSP90 PDB ids) are noteworthy and raise the question of whether *P. falciparum* HSP90 can manipulate cellular processes in human cells.

Elongation factor 1-alpha (EF-1α) is also considered a multifunctional (moonlighting) protein. EF-1α is involved in targeting and binding aminoacyl tRNA to the A-site of the ribosome and is involved in regulating cytoskeletal rearrangements [42]. *Plasmodium* EF-1α is transported by EVs and has immunosuppressive activities due to the inhibition of the CD4 + T cells’ response to antigen presentation [43]. In *Leishmania donovani*, secreted EF-1α and fructose-bisphosphate aldolase cause the activation of host macrophage protein tyrosine phosphatase and decrease the activity of these cells [44,45]. Whether or not EF-1α transported by EVs has additional roles is still unknown, and the lack of a crystallized structure makes it difficult to determine this molecule’s capacity to perform a mimetic role inside human cells.

The proteasome is a large multi-catalytic complex responsible for the degradation of intracellular proteins. The central core of the complex is the 20s proteasome, which is composed of alpha and beta subunits [46]. Among our candidates, we found a proteasome subunit alpha type (PF3D7_0727400) with structural resemblance to the same subunit type in humans. A potential explanation for this observation is that an entire proteasome is traveling through EVs to degrade *Plasmodium* proteins. This hypothesis is supported by the discovery of three proteins annotated as proteasome subunit beta (PF3D7_1470900, PF3D7_0803800, and PF3D7_1328100) in EVs, which is in line with Sampaio et al. [18] (Supplementary Table 1). The presence and role of the proteasome (or at least part of them) traveling through EVs raises an intriguing issue. Another exciting scenario is the possibility that *P. falciparum* proteasome is capable of directly degrading human proteins, although so far, no report suggesting this possibility has been published. In any case, despite the structural similarities between alpha subunits of *P. falciparum* and humans, it is hard to imagine the possibility of functional mimicry for this molecule, and a more plausible state of affairs is that convergent evolution has been acting to resemble both structures.

The family of 14-3-3 adaptor and chaperon proteins interact with signal-transduction molecules to change their activity and the subcellular localization. This kind of molecule participates in the regulation of several cellular pathways [47]. The *P. falciparum* protein 14-3-3 (PF3D7_0818200) has been observed interacting with *Pf*CDPK1 and was proposed as a target for malaria treatment [48]. However, our primary and tertiary analyses for this protein show a high resemblance with the 14-3-3 orthologue in humans (Figure 2, Supplementary Table 3). These observations discourage the use of PF3D7_0818200 as a direct target, because the similarity with human molecules could lead to a cross-reaction with a potential drug based on this molecule. The structural similarity deduced for this molecule renders PF3D7_0818200 a good candidate for performing functional mimicry.

Ribosomal protein S3 (RPS3) is part of the 40S small ribosomal subunit; surprisingly, we found remarkable similarities between this kind of protein in *P. falciparum* (PF3D7_1465900) and *Homo sapiens*. If we observe the structural similarities (Figure 2), the human RPS3 is larger and both structures coincide only in the beta sheets section and one of the several alpha helix of the human protein. Alongside the structural similarities, the question that emerges here is why a subunit of the ribosome is carried by EVs. In humans, more functions for RPS3 have been reported in addition to the ribosomal ones. For example, RPS3 was identified as part of the p65-p50 heterodimer DNA-binding complexes of the NF-kappaB transcription factor [49]. Other functions of RPS3 include the induction of apoptosis, interaction with the scanning factor DHX29, and repairing the excision damage of DNA [50]. Given that RPS3 is directly involved in DNA translation, expression, and inclusive repairing, the function for this molecule inside the ERVs is difficult to deduce without specific functional experiments.

In this work, we detected and filtered a set of proteins of *P. falciparum* found in EVs during parasite cell infection in order to detect potential mimetic molecules. To do that, we considered only complete sequences; we did not analyze epitopes or protein fractions. Furthermore, we based our search upon sequence similarities and then (only for the best results) upon structural similarities with human proteins. This approximation is limited since it can only detect mimetic proteins produced by sequence conservation or horizontal gene transfer but in the initial filter steps, it excludes the cases produced by convergent evolution. The lack of several structures of *P. falciparum* proteins makes it difficult to analyze both sequence and structure at the same time for all the candidates. At the end of all those analyses, we found that several moonlighting proteins are carried by EVs, and these have the potential to manipulate the host cells. With the methodology used in this research, we could not determine with precision if one or more of these proteins are performing functional mimicry. However, we raised several questions about the usability of moonlighting proteins of *P. falciparum* in communicating with and colonizing new cells throughout the parasite life cycle. We expect in the future that the proteins highlighted in this study can shed some light on the complex interaction between *P. falciparum* and humans.

## Supplementary Material

Supplemental Material
