# Supplementary material for: The search for molecular mimicry in proteins carried by extracellular vesicles secreted by cells infected with Plasmodium falciparum
Source: Commun Integr Biol. 2021 Sep 8;14(1):212–20. doi: 10.1080/19420889.2021.1972523 (PMC8437455; doi:10.1080/19420889.2021.1972523)
Supplement: Supplemental Material [file KCIB_A_1972523_SM4508.zip › supplementary/Supplementary Material legends.docx]

## Supplementary Material

Supplementary File 1 contains:

Supplementary Figure 1-8. Phylogenetic trees reconstructed for each candidate

**Supplementary Table 1.** *P. falciparum* proteins found in vesicles during the parasite infection in humans cells.

**Supplementary Table 2.** *P. falciparum* proteins shared by reports of EV content.

**Supplementary Table 3.** BLAST results for the search performed with *P. falciparum* candidates against *Homo sapiens* proteome.

**Supplementary Table 4.** RSMDs obtained from the superposition of candidate proteins.

**Supplementary Table 5**. InterPro and Pfam domains and motifs shared by imitator (*P. falciparum*) and imitated (*H. sapiens*) candidate proteins.
