# Supplementary material for: The search for molecular mimicry in proteins carried by extracellular vesicles secreted by cells infected with Plasmodium falciparum
Source: Commun Integr Biol. 2021 Sep 8;14(1):212–20. doi: 10.1080/19420889.2021.1972523 (PMC8437455; doi:10.1080/19420889.2021.1972523)
Supplement: Supplemental Material [file KCIB_A_1972523_SM4508.zip › supplementary/Supplementary material.docx]

**
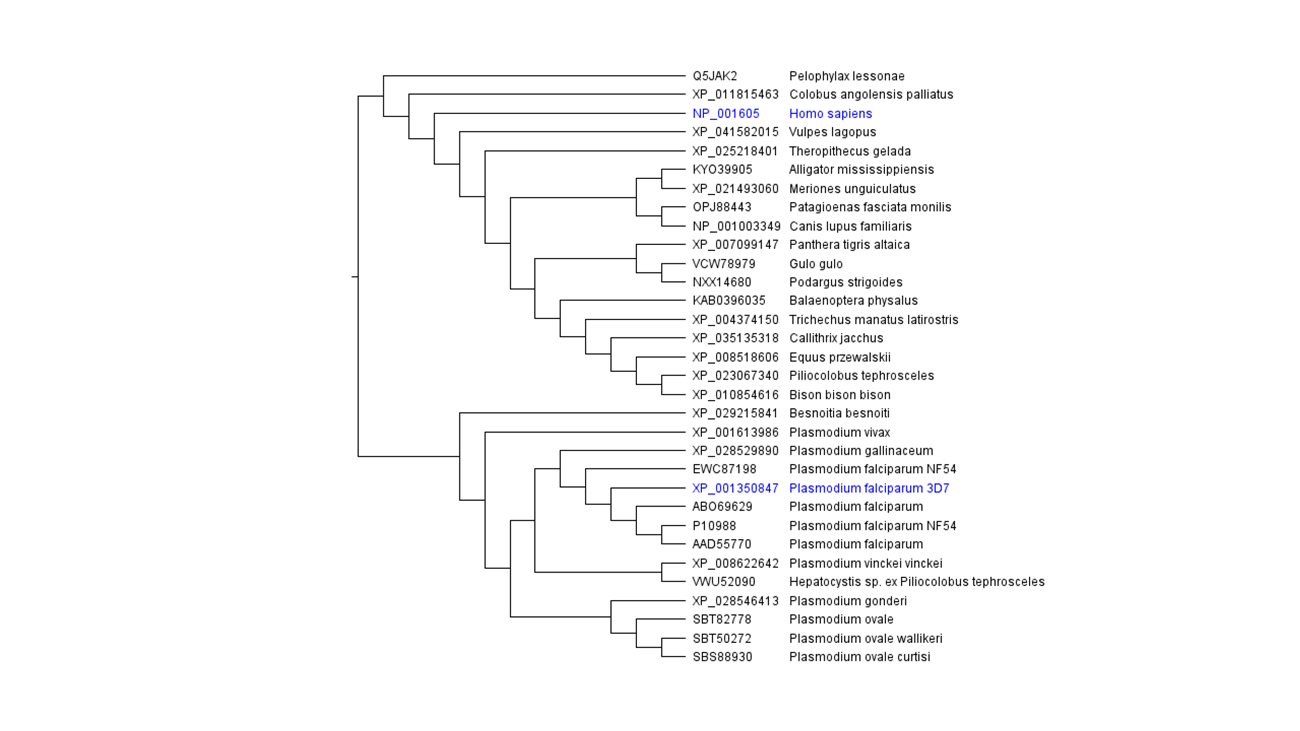
**

**Supplementary Figure 1.** Phylogenetic tree for the Actin-1. The tree was reconstructed using the most similar sequence to the putative imitator (*P. falciparum*) and imitated (*Homo sapiens*) proteins (both highlighted in blue). The similar sequences were retrieved from the NR database of NCBI.

**
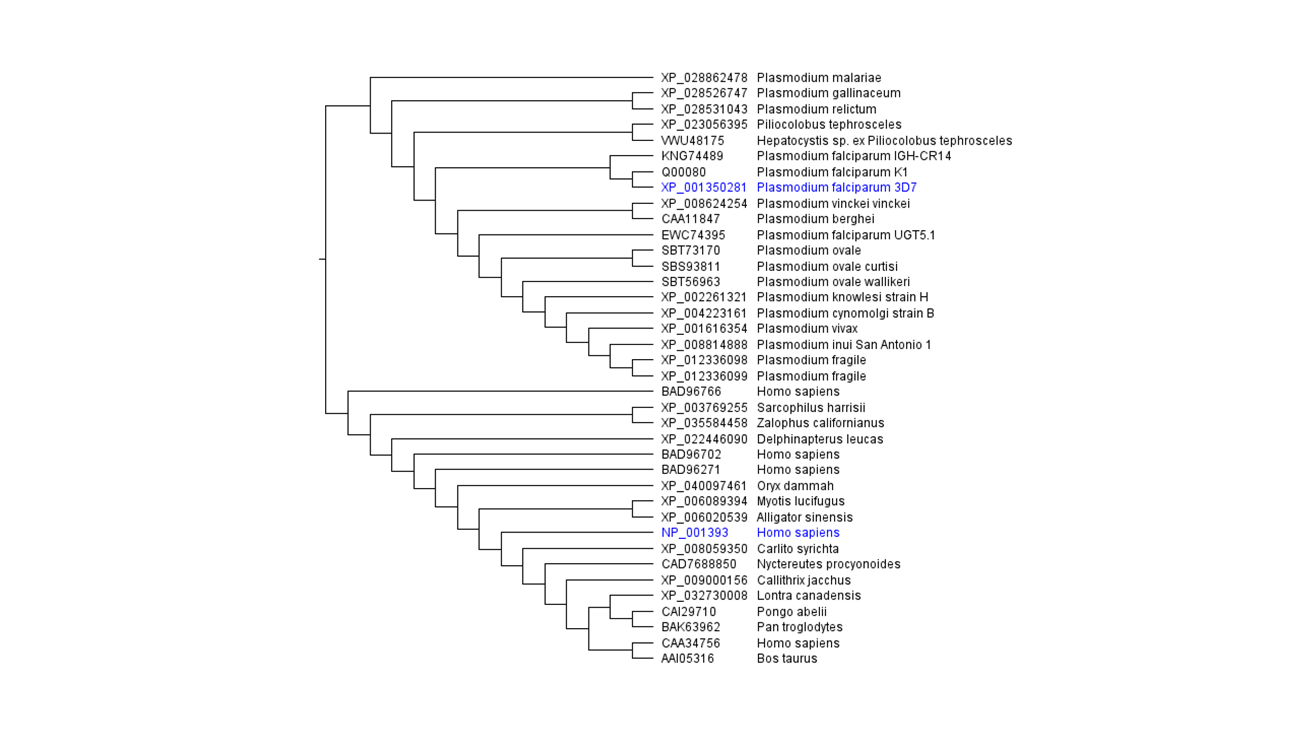
**

**Supplementary Figure 2.** Phylogenetic tree for the Elongation factor 1-alpha. The tree was reconstructed using the most similar sequence to the putative imitator (*P. falciparum*) and imitated (*Homo sapiens*) proteins (both highlighted in blue). The similar sequences were retrieved from the NR database of NCBI.

**
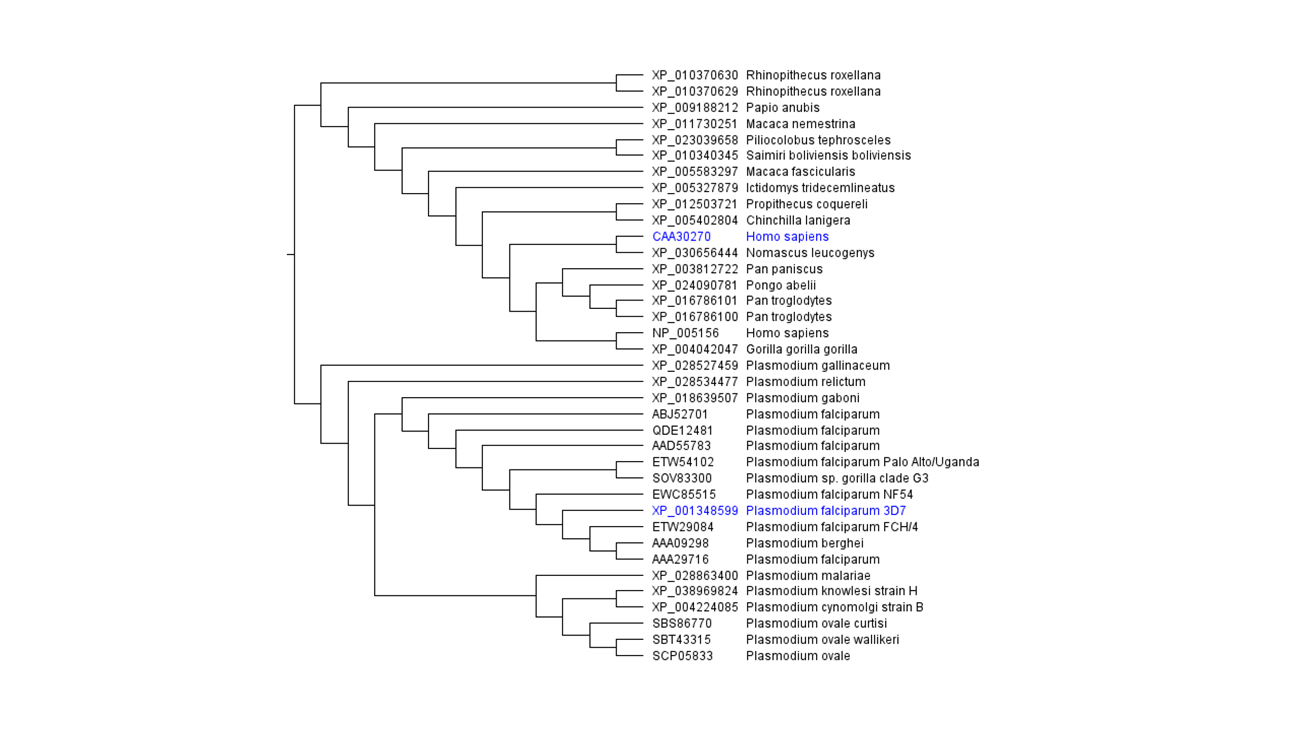
**

**Supplementary Figure 3.** Phylogenetic tree for the Fructose-bisphosphate aldolase. The tree was reconstructed using the most similar sequence to the putative imitator (*P. falciparum*) and imitated (*Homo sapiens*) proteins (both highlighted in blue). The similar sequences were retrieved from the NR database of NCBI.

**
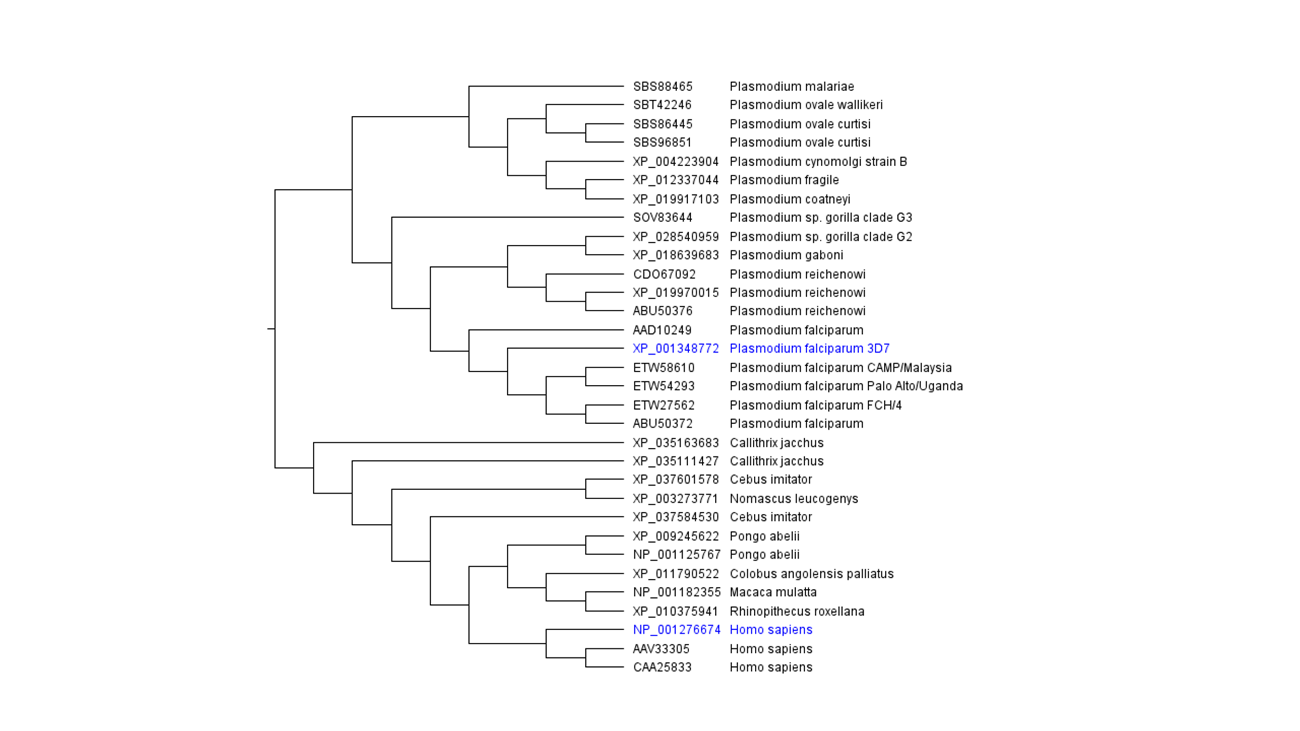
**

**Supplementary Figure 4.** Phylogenetic tree for the Glyceraldehyde-3-phosphate dehydrogenase. The tree was reconstructed using the most similar sequence to the putative imitator (*P. falciparum*) and imitated (*Homo sapiens*) proteins (both highlighted in blue). The similar sequences were retrieved from the NR database of NCBI.

**
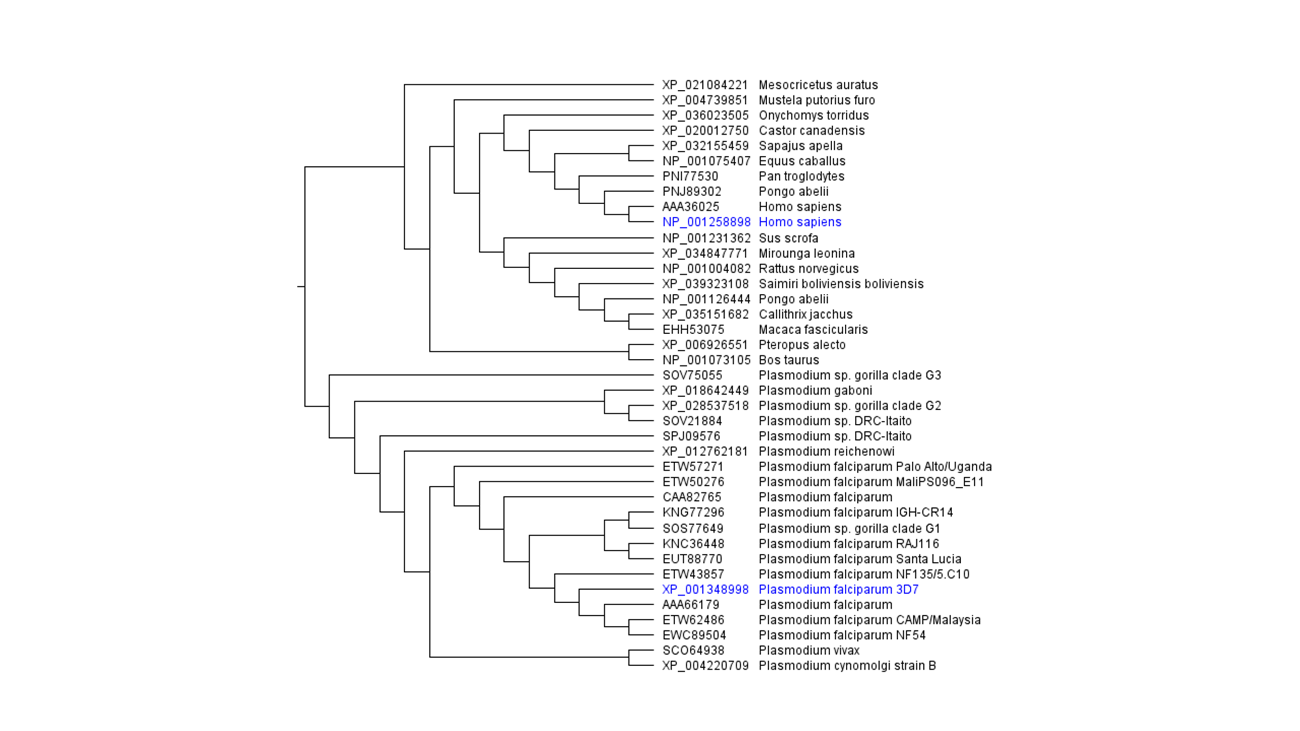
**

**Supplementary Figure 5.** Phylogenetic tree for the Heat shock protein 90. The tree was reconstructed using the most similar sequence to the putative imitator (*P. falciparum*) and imitated (*Homo sapiens*) proteins (both highlighted in blue). The similar sequences were retrieved from the NR database of NCBI.

**
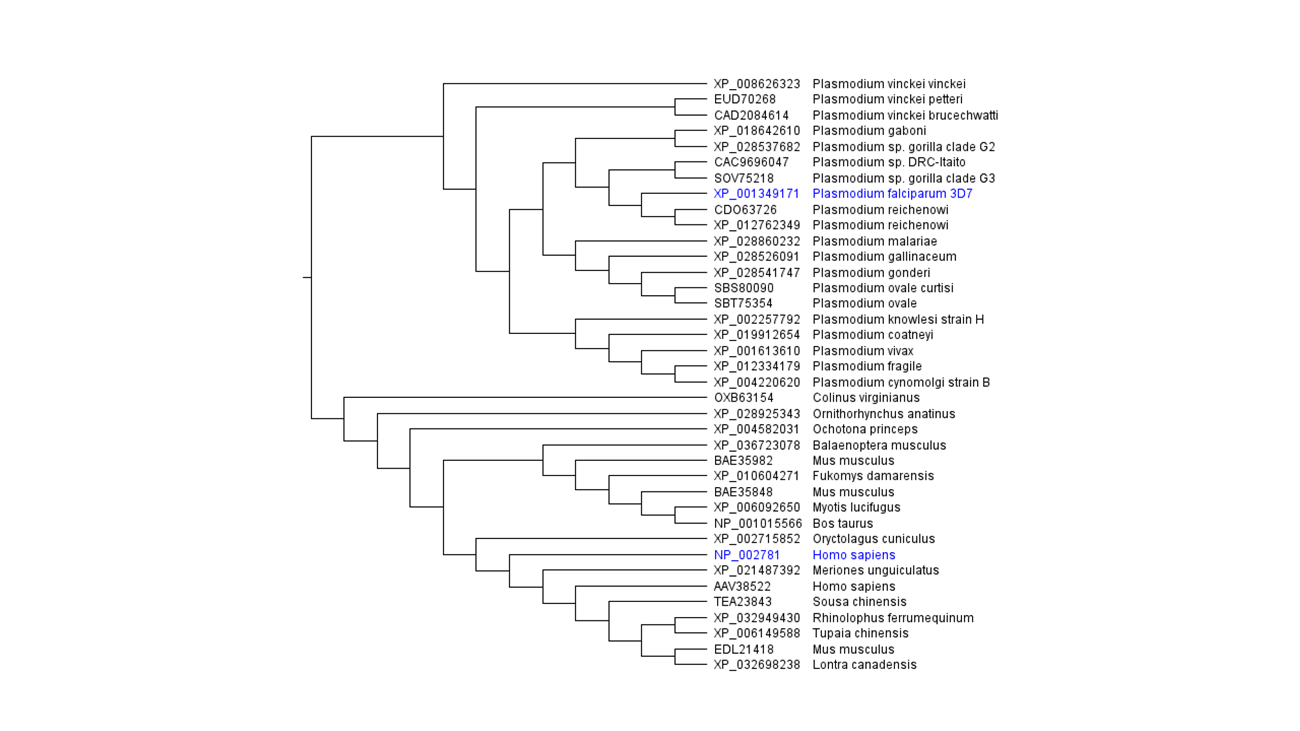
**

**Supplementary Figure 6.** Phylogenetic tree for the Proteasome subunit alpha type. The tree was reconstructed using the most similar sequence to the putative imitator (*P. falciparum*) and imitated (*Homo sapiens*) proteins (both highlighted in blue). The similar sequences were retrieved from the NR database of NCBI.


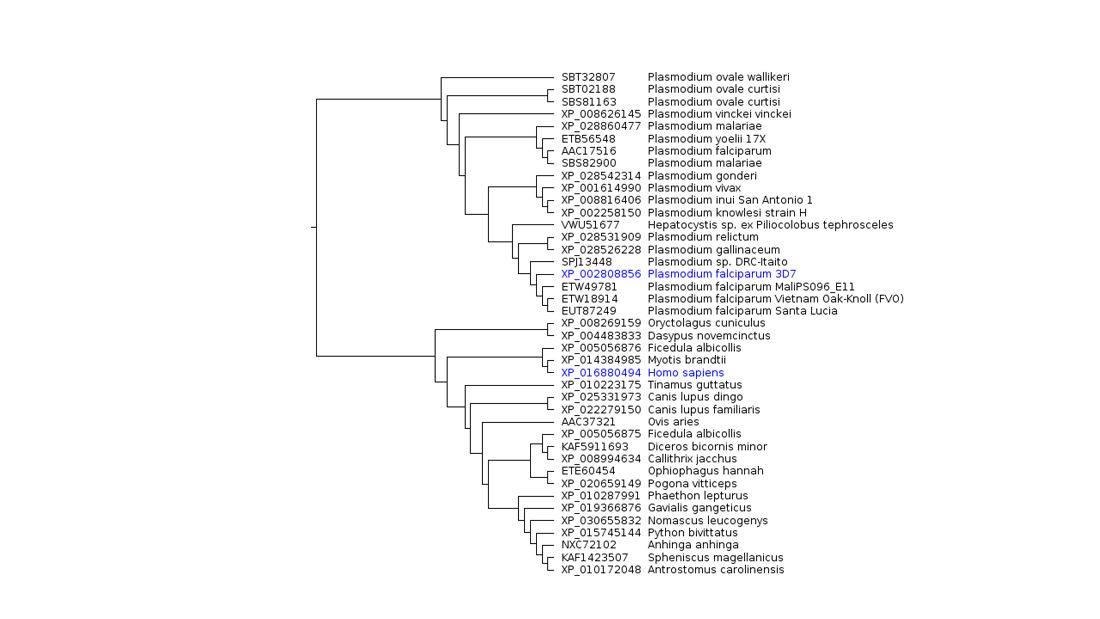


**Supplementary Figure 7.** Phylogenetic tree for the 14-3-3 protein. The tree was reconstructed using the most similar sequence to the putative imitator (*P. falciparum*) and imitated (*Homo sapiens*) proteins (both highlighted in blue). The similar sequences were retrieved from the NR database of NCBI.


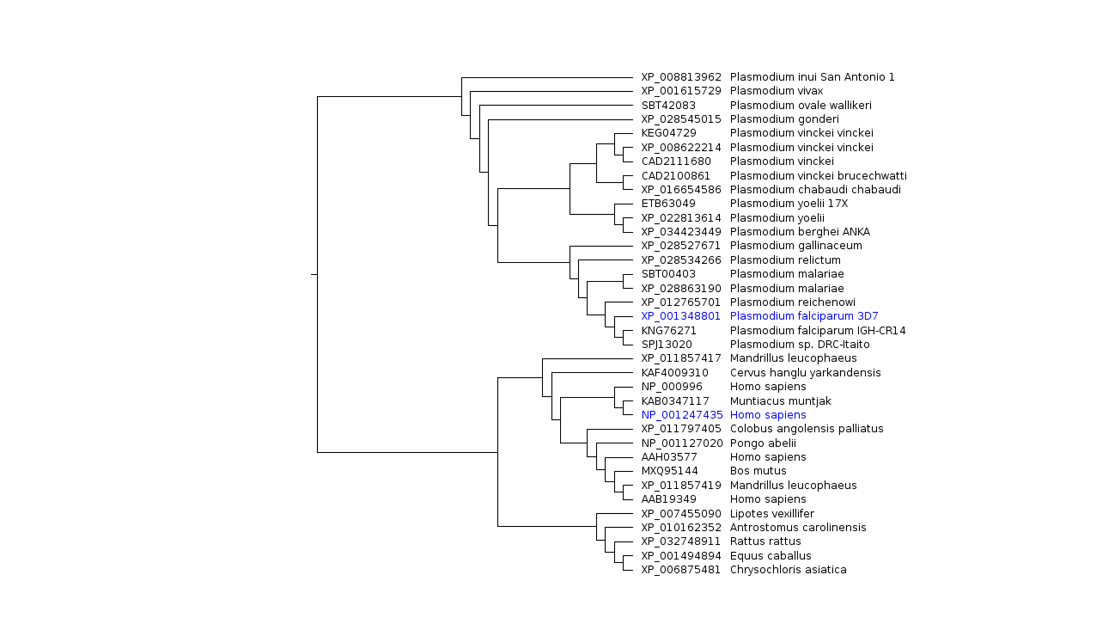


**Supplementary Figure 8.** Phylogenetic tree for the 40S ribosomal protein S3. The tree was reconstructed using the most similar sequence to the putative imitator (*P. falciparum*) and imitated (*Homo sapiens*) proteins (both highlighted in blue). The similar sequences were retrieved from the NR database of NCBI.

**Supplementary table 1.** *P. falciparum* proteins found in vesicles during the parasite infection in humans cells.

| Articles | Number of Proteins | UniProt Gene Names |
| --- | --- | --- |
| Sampaio *et al*.^1^ | 32 | PF3D7_0930300, PF3D7_0929400, PF3D7_1357100, PF3D7_0302500, PF3D7_0818900, PF3D7_0915400, PF3D7_0727400, PF3D7_0917900, PF3D7_1462800, PF3D7_0608500, PF3D7_0708400, PF3D7_0922600, PF3D7_1117700, PF3D7_1353800, PF3D7_1470900, PF3D7_0317000, PF3D7_0803800, PF3D7_0905400, PF3D7_1015600, PF3D7_1444800, PF3D7_1246200, PF3D7_1410400, PF3D7_1474800, PF3D7_0422400, PF3D7_0621200, PF3D7_0807500, PF3D7_1008700, PF3D7_1016300, PF3D7_1328100, PF3D7_1343000, PF3D7_1424100, PF3D7_1471100 |
| Mantel *et al*. ^2^ | 38 | PF3D7_0929400, PF3D7_0102500, PF3D7_0302500, PF3D7_1246200, PF3D7_0917900, PF3D7_1462800, PF3D7_1116800, PF3D7_0905400, PF3D7_1410400, PF3D7_0818900, PF3D7_1357000, PF3D7_0501600, PF3D7_0731500, PF3D7_1471100, PF3D7_0500800, PF3D7_1444800, PF3D7_0930300, PF3D7_0708400, PF3D7_1015900, PF3D7_1324900, PF3D7_1149000, PFI1105w, PF3D7_0424600, PF3D7_1370300, PF3D7_1343000, PF3D7_0102200, PF3D7_0818200, PF3D7_1436300, PF3D7_1222300, PF3D7_0626800, PF3D7_1451100, PF3D7_1108600, PF3D7_1105000, PF3D7_0501000, PF3D7_1353200, PF3D7_1465900, PF3D7_0922200, PF3D7_0501500 |
| Abdi *et al*. ^3^ | 52 | PF3D7_1462800, PF3D7_0201900, PF3D7_1357100, PF3D7_0929400, PF3D7_1246200, PF3D7_1324900, PF3D7_0935900, PF3D7_1014100, PF3D7_0202000, PF3D7_0702500, PF3D7_0702400, PF3D7_1410400, PF3D7_1116800, PF3D7_1343000, PF3D7_0930300, PF3D7_1117700, PF3D7_0818900, PF3D7_1353100, PF3D7_1468700, PF3D7_0601900, PF3D7_1347500, PF3D7_0826700, PF3D7_1035300, PF3D7_0501600, PF3D7_1471100, PF3D7_0302500, PF3D7_1252100, PF3D7_0621200, PF3D7_0422400, PF3D7_0207600, PF3D7_0322900, PF3D7_1465900, PF3D7_0301700, PF3D7_0801000, PF3D7_0831700, PF3D7_1353200, PF3D7_0814200, PF3D7_1130200, PF3D7_1424100, PF3D7_1108400, PF3D7_0424600, PF3D7_1035700, PF3D7_1105400, PF3D7_0918000, PF3D7_0818200, PF3D7_1219100, PF3D7_1447000, PF3D7_0501000, PF3D7_1370300, PF3D7_0727400, PF3D7_1361800, PF3D7_0501500 |

**Supplementary Table 2.** *P. falciparum* proteins shared by reports of EV content

| **Articles** | **Number of shared proteins** | UniProt Gene Names |
| --- | --- | --- |
| Sampaio et al. (2018), Mantel et al. (2013),  Abdi et al. (2017) | 9 | PF3D7_1343000 PF3D7_0930300 PF3D7_1471100 PF3D7_0302500 PF3D7_0929400 PF3D7_1410400 PF3D7_1462800 PF3D7_1246200 PF3D7_0818900 |
| Abdi et al. (2017),  Mantel et al. (2013) | 10 | PF3D7_0424600 PF3D7_1116800 PF3D7_1324900 PF3D7_0501600 PF3D7_0501500 PF3D7_1353200 PF3D7_0818200 PF3D7_0501000 PF3D7_1465900 PF3D7_1370300 |
| Sampaio et al. (2018),  Abdi et al. (2017) | 6 | PF3D7_0727400 PF3D7_1357100 PF3D7_0621200 PF3D7_0422400 PF3D7_1117700 PF3D7_1424100 |
| Sampaio et al. (2018),  Mantel et al. (2013) | 4 | PF3D7_0917900 PF3D7_0905400 PF3D7_0708400 PF3D7_1444800 |
| Mantel et al. (2013) | 15 | PF3D7_0626800 PF3D7_0922200 PF3D7_0102500 PF3D7_1108600 PF3D7_1451100 PF3D7_1436300 PFI1105w PF3D7_1105000 PF3D7_1149000 PF3D7_0500800 PF3D7_1222300 PF3D7_0731500 PF3D7_1015900 PF3D7_0102200 PF3D7_1357000 |
| Sampaio et al. (2018) | 13 | PF3D7_0803800 PF3D7_1470900 PF3D7_1474800 PF3D7_1328100 PF3D7_0922600 PF3D7_1015600 PF3D7_0317000 PF3D7_0915400 PF3D7_1008700 PF3D7_0807500 PF3D7_1353800 PF3D7_1016300 PF3D7_0608500 |
| Abdi et al. (2017) | 27 | PF3D7_1353100 PF3D7_0301700 PF3D7_0702400 PF3D7_0826700 PF3D7_1130200 PF3D7_1447000 PF3D7_1105400 PF3D7_1252100 PF3D7_1035300 PF3D7_0207600 PF3D7_1347500 PF3D7_0702500 PF3D7_0202000 PF3D7_1035700 PF3D7_1219100 PF3D7_0201900 PF3D7_0601900 PF3D7_1468700 PF3D7_0801000 PF3D7_0935900 PF3D7_1014100 PF3D7_1108400 PF3D7_0918000 PF3D7_0831700 PF3D7_0322900 PF3D7_1361800 PF3D7_0814200 |

**Supplementary Table 3.** BLAST results for the search performed with *P. falciparum* candidates against *Homo sapiens* proteome.

| ***P. falciparum***  **Sequence ID** | ***Homo sapiens***  **Sequence ID** | **BLASTp results** | | | |
| --- | --- | --- | --- | --- | --- |
|  |  | **% Identity** | **Query Coverage** | **Length** | **E-value** |
| PF3D7_1246200 | NP_001605.1 | 82,62 | 99 | 374 | 0 |
| PF3D7_0818900 | NP_694881.1 | 77,074 | 68 | 458 | 0 |
| PF3D7_0708400 | NP_031381.2 | 74,766 | 29 | 214 | 1,77E-101 |
| PF3D7_1117700 | NP_006316.1 | 72,539 | 90 | 193 | 3,36E-104 |
| PF3D7_1357100 | NP_001393.1 | 68,928 | 100 | 457 | 0 |
| PF3D7_0818200 | XP_016880494.1 | 66,83 | 78 | 205 | 1,74E-97 |
| PF3D7_0917900 | NP_005338.1 | 66,667 | 96 | 630 | 0 |
| PF3D7_1465900 | NP_001247435.1 | 65,24 | 98 | 233 | 6,9E-101 |
| PF3D7_1462800 | NP_002037.2 | 64,286 | 99 | 336 | 2,96E-159 |
| PF3D7_1370300 | XP_016855889.1 | 56,25 | 13 | 32 | 0,54 |
| PF3D7_1444800 | NP_005156.1 | 55,89 | 97 | 365 | 6,9E-132 |
| PF3D7_0727400 | NP_002781.2 | 54,357 | 94 | 241 | 6,58E-90 |
| PF3D7_0501500 | NP_055391.2 | 52 | 6 | 25 | 7,5 |
| PF3D7_0930300 | NP_003794.3 | 44,615 | 4 | 65 | 0,18 |
| PF3D7_0501600 | NP_001291420.1 | 43,478 | 6 | 23 | 7,5 |
| PF3D7_1324900 | NP_001180565.1 | 40,82 | 15 | 49 | 2 |
| PF3D7_1471100 | NP_001121364.1 | 40,625 | 11 | 32 | 4,2 |
| PF3D7_0422400 | NP_001351619.1 | 39,02 | 24 | 41 | 0,91 |
| PF3D7_1116800 | XP_011521972.1 | 37,5 | 5 | 48 | 0,56 |
| PF3D7_1353200 | NP_705933.2 | 32,653 | 36 | 49 | 5,3 |
| PF3D7_1343000 | NP_940892.1 | 31,71 | 80 | 164 | 5,09E-27 |
| PF3D7_1410400 | NP_001073964.2 | 31,03 | 11 | 87 | 2,4 |
| PF3D7_0905400 | XP_016867101.1 | 30,909 | 12 | 110 | 0,58 |
| PF3D7_1424100 | NP_001336409.1 | 28,42 | 32 | 95 | 4,2 |
| PF3D7_0501000 | XP_016860977.1 | 28 | 38 | 100 | 0,15 |
| PF3D7_0302500 | NP_001138381.1 | 26,88 | 6 | 93 | 4 |
| PF3D7_0424600 | NP_001308725.1 | 26,05 | 37 | 119 | 0,62 |
| PF3D7_0621200 | NP_004478.3 | 24,03 | 49 | 154 | 0,76 |
| PF3D7_0929400 | NP_877963.1 | 23,622 | 9 | 127 | 6,8 |

**Supplementary Table 4.** RSMDs obtained from the superposition of candidate proteins.

| **UniProt ID** | **Gene name** | **PDB**  ***P. falciparum*** | **PDB**  ***Homo sapiens*** | **RMSD** |
| --- | --- | --- | --- | --- |
| A0A144A1R5 | PF3D7_1246200 | 6I4K | 5JLH | 1.532 |
| Q8IB24 | PF3D7_0818900 | Predicted structure | 5AQM | 3.671 |
| Q8IC05 | PF3D7_0708400 | 3K60 | 5FWK | 5.979 |
| Q7KQK6 | PF3D7_1117700 | Predicted structure | 6A3A | 1.485 |
| Q8I0P6 | PF3D7_1357100 | Predicted structure | 3C5J | 5.232 |
| C0H4V6 | PF3D7_0818200 | Predicted structure | 3UBW | 0.580 |
| Q8I2X4 | PF3D7_0917900 | Predicted structure | 6ASY | 2.519 |
| Q8IKH8 | PF3D7_1465900 | 6OKK | 6YBS | 0.76 |
| Q8IKK7 | PF3D7_1462800 | 1YWG | 3GPD | 1.277 |
| A0A144A3T1 | PF3D7_1444800 | 2PC4 | 1XFB | 0.876 |
| Q8IBI3 | PF3D7_0727400 | 6MUW | 6REY | 1.664 |

**Supplementary Table 5**. InterPro and Pfam domains and motifs shared by imitator (*P. falciparum*) and imitated (*H. sapiens*) candidate proteins

| **Pfam ID** | **Name** | **Interpro ID** | **Name** |  |
| --- | --- | --- | --- | --- |
| **Fructose-bisphosphate aldolase** | | | | |
| PF00274 | Fructose-bisphosphate aldolase class-I | IPR000741 | Fructose-bisphosphate aldolase, class-I |  |
|  |  | IPR013785 | Aldolase-type TIM barrel |  |
|  |  | IPR029768 | Fructose-bisphosphate aldolase class-I active site |  |
| **Actin-1** | | | | |
| PF00022 | Actin | IPR004000 | Actin family |  |
|  |  | IPR004001 | Actin, conserved site |  |
|  |  | IPR043129 | ATPase, nucleotide binding domain |  |
|  |  | IPR020902 | Actin/actin-like conserved site |  |
| **Elongation factor 1-alpha** | | | | |
| PF00009 | Elongation factor Tu GTP binding domain | IPR000795 | Translational (tr)-type GTP-binding domain |  |
| PF03144 | Elongation factor Tu domain 2 | IPR004160 | Translation elongation factor EFTu/EF1A, C-terminal |  |
| PF03143 | Elongation factor Tu C-terminal domain | IPR004161 | Translation elongation factor EFTu-like, domain 2 |  |
|  |  | IPR004539 | Translation elongation factor EF1A, eukaryotic/archaeal |  |
|  |  | IPR009000 | Translation protein, beta-barrel domain superfamily |  |
|  |  | IPR009001 | Translation elongation factor EF1A/initiation factor IF2gamma, C-terminal |  |
|  |  | IPR027417 | P-loop containing nucleoside triphosphate hydrolase |  |
|  |  | IPR031157 | Tr-type G domain, conserved site |  |
| **Glyceraldehyde-3-phosphate dehydrogenase** | | | | |
| PF02800 | Glyceraldehyde 3-phosphate dehydrogenase, C-terminal domain | IPR020831 | Glyceraldehyde/Erythrose phosphate dehydrogenase family |  |
| PF00044 | Glyceraldehyde 3-phosphate dehydrogenase, NAD binding domain | IPR036291 | NAD(P)-binding domain superfamily |  |
|  |  | IPR020830 | Glyceraldehyde 3-phosphate dehydrogenase, active site |  |
|  |  | IPR020829 | Glyceraldehyde 3-phosphate dehydrogenase, catalytic domain |  |
|  |  | IPR020828 | Glyceraldehyde 3-phosphate dehydrogenase, NAD(P) binding domain |  |
|  |  | IPR006424 | Glyceraldehyde-3-phosphate dehydrogenase, type I |  |
| **Heat shock protein 90** | | | | |
| PF00183 | Hsp90 protein | IPR037196 | HSP90, C-terminal domain |  |
| PF02518 | Histidine kinase-, DNA gyrase B-, and HSP90-like ATPase | IPR001404 | Heat shock protein Hsp90 family |  |
|  |  | IPR003594 | Histidine kinase/HSP90-like ATPase |  |
|  |  | IPR019805 | Heat shock protein Hsp90, conserved site |  |
|  |  | IPR036890 | Histidine kinase/HSP90-like ATPase superfamily |  |
|  |  | IPR020568 | Ribosomal protein S5 domain 2-type fold |  |
|  |  | IPR020575 | Heat shock protein Hsp90, N-terminal |  |
| **Proteasome subunit alpha** | | | | |
| PF00227 | Proteasome subunit | IPR029055 | Nucleophile aminohydrolases, N-terminal |  |
|  |  | IPR000426 | Proteasome alpha-subunit, N-terminal domain |  |
|  |  | IPR001353 | Proteasome, subunit alpha/beta |  |
|  |  | IPR033812 | Proteasome subunit alpha5 |  |
|  |  | IPR023332 | Proteasome alpha-type subunit |  |
| **14-3-3 protein I** | | | | |
| PF00244 | 14-3-3 protein | IPR023410 | 14-3-3 domain |  |
|  |  | IPR000308 | 14-3-3 protein |  |
|  |  | IPR023409 | 14-3-3 protein, conserved site |  |
|  |  | IPR036815 | 14-3-3 domain superfamily |  |
| **40S ribosomal protein S3** | | | | |
| PF00189 | Ribosomal protein S3, C-terminal domain | IPR001351 | Ribosomal protein S3, C-terminal |  |
| PF07650 | KH domain | IPR036419 | Ribosomal protein S3, C-terminal domain superfamily |  |
|  |  | IPR009019 | K homology domain superfamily, prokaryotic type |  |
|  |  | IPR004044 | K Homology domain, type 2 |  |
|  |  | IPR015946 | K homology domain-like, alpha/beta |  |

**References**

1. Sampaio NG, Emery SJ, Garnham AL, Tan QY, Sisquella X, Pimentel MA, Jex AR, Regev-Rudzki N, Schofield L, Eriksson EM. Extracellular vesicles from early stage *Plasmodium falciparum* -infected red blood cells contain PfEMP1 and induce transcriptional changes in human monocytes. Cellular Microbiology 2018; 20:e12822.

2. Mantel P-Y, Hoang AN, Goldowitz I, Potashnikova D, Hamza B, Vorobjev I, Ghiran I, Toner M, Irimia D, Ivanov AR, et al. Malaria-Infected Erythrocyte-Derived Microvesicles Mediate Cellular Communication within the Parasite Population and with the Host Immune System. Cell Host & Microbe 2013; 13:521–34.

3. Abdi A, Yu L, Goulding D, Rono MK, Bejon P, Choudhary J, Rayner J. Proteomic analysis of extracellular vesicles from a *Plasmodium falciparum* Kenyan clinical isolate defines a core parasite secretome. Wellcome open research 2017; 2:50.
